# Evaluation of a biosensor-based graphene oxide-DNA nanohybrid for lung cancer

**DOI:** 10.1039/d2ra05808a

**Published:** 2023-01-17

**Authors:** Mustafa M. Kadhim, Ahmed Mahdi Rheima, Zainab S. Abbas, Haider Hussain Jlood, Safa K. Hachim, Wesam R. Kadhum, Ehsan kianfar

**Affiliations:** a Medical Laboratory Techniques Department, Al-Farahidi University Baghdad 10022 Iraq; b Department of Chemistry, College of Science, Mustansiriyah University Baghdad Iraq; c Research Center, The University of Mashreq 10021 Baghdad Iraq; d Pharmacy Department, Mazaya University College Thi-Qar Iraq; e College of Technical Engineering, The Islamic University Najaf Iraq; f Medical Laboratory Techniques Department, Al-Turath University College Iraq Baghdad; g Department of Pharmacy, Kut University College Kut 52001 Wasit Iraq; h Istanbul Medeniyet University Istanbul Turkey ehsan_kianfar2010@yahoo.com +90 917-744-1049; i Department of Chemical Engineering, Islamic Azad University Arak Branch Arak Iran; j Young Researchers and Elite Club, Islamic Azad University Gachsaran Branch Gachsaran Iran; k Department of Chemistry, Islamic Azad University Sousangerd Branch Sousangerd Iran

## Abstract

Lung cancer is nowadays among the most prevalent diseases worldwide and features the highest mortality rate among various cancers, indicating that early diagnosis of the disease is of paramount importance. Given that the conventional methods of cancer detection are expensive and time-consuming, special attention has been paid to the provision of less expensive and faster techniques. In recent years, the dramatic advances in nanotechnology and the development of various nanomaterials have led to activities in this context. Recent studies indicate that the graphene oxide (GO) nanomaterial has high potential in the design of nano biosensors for lung cancer detection owing to its unique properties. In the current article, a nano biosensor based on a DNA-GO nanohybrid is introduced to detect deletion mutations causing lung cancer. In this method, mutations were detected using a FAM-labeled DNA probe with fluorescence spectrometry. GO was synthesized according to Hummers' method and examined and confirmed using Fourier Transform Infrared (FT-IR) Spectrometry and UV-vis spectrometry methods and Transmission Electron Microscopy (TEM) images.

## Abstract 2

Lung cancer is nowadays among the most prevalent diseases worldwide and features the highest mortality rate among various cancers, indicating that early diagnosis of the disease is of paramount importance.

## Graphical abstract

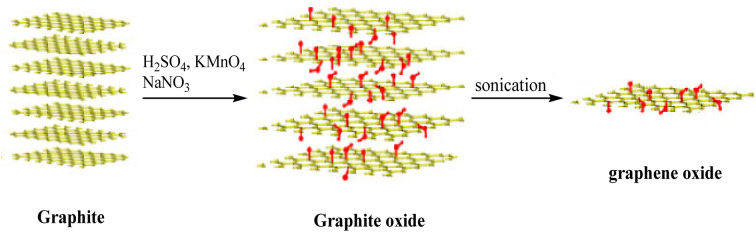

## 1. Introduction

Cancer is a genetic disease that results from the uncontrolled growth and division of cells in a part of the body that results from environmental factors and genetic disorders.^1–5^ In other words, cancer occurs as a result of a series of mutations in human genes.^6–10^ There are more than 200 types of cancer today, one of the most common of which is lung cancer.^11–15^

Lung cancer is the second most common cancer in men and women and is one of the most preventable cancers. There are generally two types of lung cancer:^16–21^

(1) Small cell lung cancer (SCLC)

(2) Non-small cell lung cancer (NSCLC)

How both grow and spread in the body and how to treat them are different. Lung cancers are classified under the microscope based on the appearance of the cells. Non-small cell lung cancer (NSCLC) is also divided into three categories:^22–25^

(1) Superficial tissue cancer, (2) mucosal and lymph node carcinoma (glandular epithelium), and (3) large cell lung cancer.^26–30^ Among people with this type of cancer, about 85–90% of cases are NSCLC and about 10–15% of cases are SCLC.

The most common clinical symptoms of lung cancer include persistent and chronic cough, chest pain, anorexia, weight loss, sputum, shortness of breath, respiratory infections such as bronchitis, the onset of wheezing and … is, which usually does not appear in the early stages of the disease. Therefore, the mortality rate of this type of cancer is very high.^31–34^ The most prevalent symptoms of lung cancer include continuous and chronic cough, thoracic pain, anorexia, weight loss, hemoptysis, dyspnea, and respiratory infections such as bronchitis, the onset of wheezing, *etc.*, which do not typically appear in the early stages of the disease, leading to a high mortality rate in this type of cancer.^35–38^

In the field of medicine, lung cancer has so far been detected using various methods, including chest radiography (x-ray), computed tomography (CT) scan, magnetic resonance imaging (MRI), bone scan, bronchoscopy, and sputum cytology.^39–43^ In recent years, the dramatic advances in nanotechnology and the development of various nanomaterials have facilitated the detection of cancer biomarkers with high accuracy and sensitivity.^44–47^ Nanotechnology has provided faster, less expensive, and easier methods with lower detection limits for lung cancer detection. Nanomaterials used in these methods include silica nanowires, gold nanoparticles, carbon nanotubes, quantum dots, magnetic nanoparticles, *etc.*^48–52,102–106^

Biomarkers are an indicator of the disease biological condition that are used to detect the disease. These biomarkers are also used to study cellular processes and to recognize and control cessation, or changes in the cellular processes of cancer cells.^53–57,107–111^ Biomarkers can be proteins, mutated DNA, RNA, lipids, carbohydrates, and small molecules resulting from cellular metabolism.^58–60,112–118^Table 1 exemplifies some of these biomarkers.^14^

**Table 1:** Biomarkers used in the detection of various cancers^14^

Biomarker	Type of cancer
PSA, PSMA	Prostate
CEA, NSE	Lung
CA 19-9, BTA	Pancreas
NMP22, BTA	Bladder
CA 15-3, 27, 29, Her-2/neu	Breasts

## 2. Materials and experimental

Materials and solvents used in this work along with the manufacturers' names were graphite powder (Merck), 10% hydrochloric acid (Merck), P_2_O_5_ (Merck), potassium chloride (AppliChem), potassium persulfate (Merck), concentrated sulfuric acid (Merck), magnesium chloride (Scharlau), sodium chloride (Scharlau), Tris (hydroxymethyl) aminomethane (Merck), 30% hydrogen peroxide (Merck), potassium permanganate (Dr Mojallali Chemical Laboratories), distilled water, and sterile water. Table 2 represents the nucleotide sequences of the oligonucleotides (Takapouzist Co.) including DNA probes, target DNA, and mDNA.^61–63^

**Table 2:** The nucleotide sequences of DNAs used in this study^a^

Oligonucleotide	Sequence
DNA probe	5′-FAM-CCTGTTGCTTCTCTTAATTCC–NH_2_–3′
Target DNA	5′-GGAATTAAGAGAAGCAACAGG-3′
mDNA (mismatched DNA)	CCATCATAGTGTCCTCCTGAACC-3′-5′

a (A) Adenine, (T) thymine, (C) cytosine, (G) guanine.

In the present work, transmission electron microscopic (TEM) images were prepared with a it should be performed JEOL JEM 2010Fas device (at 200 kV). The UV-vis and the IR KBr pellet spectra were respectively obtained using Shimadzu 1800 and PerkinElmer Spectrum Version 10.4.00 and Jasco Fp-750 spectrometers. Fluorescence spectra were determined with a Jasco Fp (Table 3).

**Table 3:** Devices used in this study

Device	Manufacturer
5810 R centrifuge	Eppendorf
pH meter	Metrohm
Sampler	Transferpette

### 2.1. Preparation of the Tris–HCl buffer

Tris(hydroxymethyl)amino methane (0.242 g) was first dissolved in an Erlenemayer containing 100 ml of sterile water, and the solution pH was made to 7.4 using HCl (with a pH meter). Then, the Tris–HCl buffer was obtained with adding 0.5844 g of sodium chloride, 0.0373 g of potassium chloride, and 0.102 g of magnesium chloride to this solution.^64–68^

### 2.2. Synthesis of GO

To synthesize GO with Hummer's method, 0.5 g P_2_O_5_, 0.5 g potassium persulfate, and 0.5 g graphite powder were poured into a beaker containing concentrated H_2_SO_4_ and incubated at 80 °C for 6 h. The solution was then diluted with 50 ml distilled water and filtered afterward. The filter paper content was washed with 50 ml distilled water and fully dried at the ambient temperature overnight. Then, 0.25 g of the resulting powder was poured into a beaker containing 11.5 ml of concentrated H_2_SO_4_ in an ice bath, followed with adding 1.5 g of KMnO_4_ while stirring continuously. Sodium nitrate was added after 15 min and stirred at 35 °C for 2 h. Next, 25 ml of distilled water was added to the reaction solution and stirred for 15 min. The reaction was stopped with adding 75 ml of distilled water and 2 ml of H_2_O_2_ (30%), yielding a yellow solution. This solution was centrifuged and the obtained pellet was washed with HCL (10%) and then with distilled water several times to completely remove the existing acids and metal ions.^69,70,101^ The graphite oxide solution was ultrasonicated for 10 min and then centrifuged at 1000 rpm for 10 min, which was repeated several times to obtain the GO solution.

### 2.3. Preparation of solutions for the fluorescence spectrum measurement

The fluorescence spectra were drawn in six steps, namely (1) drawing the DNA probe fluorescence spectrum, (2) Optimizing DNA probe adsorption time on the GO surface, (3) optimizing the GO dose in the presence of the DNA probe, (4) optimizing the target DNA hybridization time with the DNA probe in the presence of GO, (5) drawing the fluorescence spectrum of the DNA-GO probe complex at different concentrations of target DNA, and (6) drawing the fluorescence spectrum of GO-DNA probe in the presence of mDNA. The fluorescence intensities of the solutions were measured in all the above-mentioned steps with an excitation wavelength of 485 nm.^71–73^

#### 2.3.1. Preparation of the first-step solution

First, 10 μl of a 10^4^ nM solution of the DNA probe was made into a final volume of 2 ml using the Tris–HCl buffer. The solution was vortexed and underwent emission spectrum measurement.^74–76^

#### 2.3.2. Preparation of the second-step solution

First, 10 μl of the 10^4^ nM solution of the DNA probe and 15 ml of a 1 mg ml^−1^ GO solution were made into a final volume of 2 ml using the Tris–HCl buffer. The solution was vortexed and underwent emission spectrum measurements at 1, 3, 5, 7, 10, 12, 14, 16, 18, 20, 22, 24, 26, 28, 30, 32, 34, and 36 min.^77–79^

#### 2.3.3. Preparation of the third-step solution

In this step, the 10^4^ nM solution of the DNA probe was poured into six prepared solutions, each of which received known volumes (10, 20, 30, 35, and 40 μl) of the 1 mg ml^−1^ GO solution. Each solution was then made into a final volume of 2 ml using the Tris–HCl buffer, followed with vortexing and undergoing fluorescence emission spectral measurements after the optimal time of the DNA probe adsorption on the GO surface.^80–82^

#### 2.3.4. Preparation of the fourth-step solution

First, 10 μl of the 104 nM solution of the DNA probe and 35 ml of a 1 mg ml^−1^ GO solution were poured into a known amount of the Tris–HCl buffer. After the optimal time of the DNA probe adsorption on the GO surface, 10 μl of the 104 nM solution of the target DNA was added to make the solution into a total volume of 2 ml. The solution was vortexed and underwent emission spectrum measurements at 2, 5, 30, 34, 36, and 42 min.^83–86^

#### 2.3.5. Preparation of the fifth-step solution

In this step, 10 μl of 10^4^ nM solution of the DNA probe and 35 ml of the 1 mg ml^−1^ GO solution were poured into five prepared solutions, each of which received a known volume of the Tris–HCl buffer. After the optimal time of the DNA probe adsorption on the GO surface, each solution was then made into a final volume of 2 ml using the 10^4^ nM solution of the target DNA at different volumes (2.5, 5, 7.5, 10, and 15 μl). The solutions were vortexed and their fluorescence emission spectra were measured after the optimal hybridization time of the DNA probe and target DNA.^87,88^

#### 2.3.6. Preparation of the sixth-step solutions

First, 10 μl of the 10^4^ nM solution of the DNA probe and 35 ml of the 1 mg ml^−1^ GO solution were poured into a known amount of the Tris–HCl buffer. After the optimal time of the DNA probe adsorption on the GO surface, 10 μl of the 10^4^ nM solution of mDNA was added to make the solution into a total volume of 2 ml. The solution was then vortexed and underwent emission spectrum measurements after the optimal hybridization time of the DNA probe and target DNA.^89^

## 3. Results and discussion

### 3.1. Preparation of GO from graphite

**Fig. 1:**
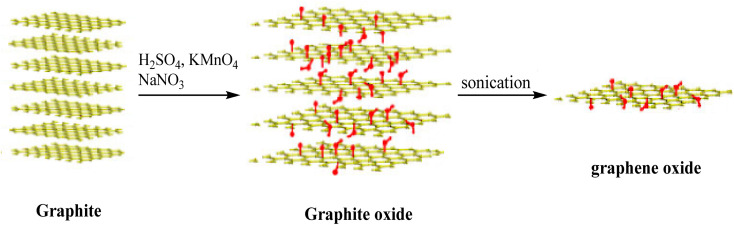
Conversion of graphite to GO.

GO was synthesized using graphite powder in the presence of concentrated H_2_SO_4_, NaNO_3_, and KMnO_4_ according to the Hummers' method (Fig. 1).^48^

### 3.2. Examination of the GO UV-vis spectrum

**Fig. 2:**
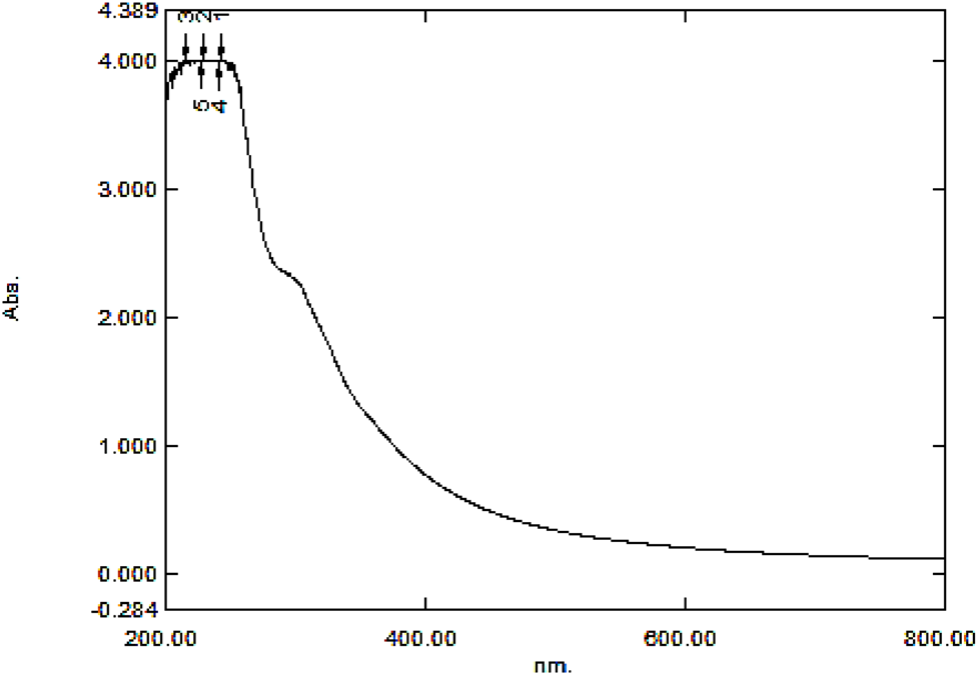
GO UV-vis spectrum.

The structure of the GO synthesized with the Hummers' method was examined and confirmed using the GO absorption UV-vis spectrum (Fig. 2), which corresponds to those reported in previous studies. The strong and weak bands appearing in 230 and 300 nm wavelengths are respectively attributed to π–π* and n–π* transitions of carbonyl groups.^49,50^

### 3.3. Interpretation of the GO IR spectrum

**Fig. 3:**
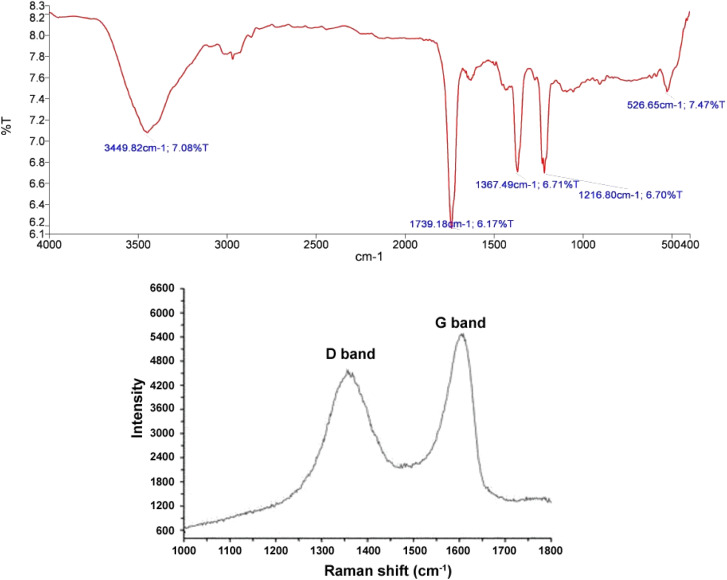
The graphene oxide IR spectrum. (a) Raman spectrum of graphene oxide.

The absorption band observed in the 3449 cm^−1^ region belongs to OH stretching vibrations. The weak absorption bands in 2867 and 2927 cm^−1^ regions are related to the CH of aldehyde groups. The absorption bands appearing in 1633 and 1065 cm^−1^ regions correspond to C=C and C–O stretching vibrations, respectively. The absorption band emerged in the 875 cm^−1^ region belongs to CH_Ar_ out-of-plane bending vibrations.^91–93^ Since the absorption band of the acidic C=O group is not observed in the 1730 cm^−1^ region, the synthesized graphene oxide contains lower carboxylic acid groups and possesses mostly alcoholic and aldehyde groups(Fig. 3). Furthermore, Fig. 3(a) illustrates the Raman spectum of graphene oxide includes D and G peaks where the D peak at ∼1350 (cm^−1^) is the result of defects in the Graphene sheets and the G peak at ∼1600 (cm^−1^) is the result of bond stretching of sp^2^ hybridized Carbons, respectively.^56,57^

### 3.4. Examination of the GO TEM image

Sample preparation methods TEM can be divided into two general categories. The first category are methods that include reducing the thickness of the sample by chemical or mechanical methods until a thin sample remains. The second category are methods that involve cutting the sample along the crystal planes to obtain a very thin section of the sample.

**Fig. 4:**
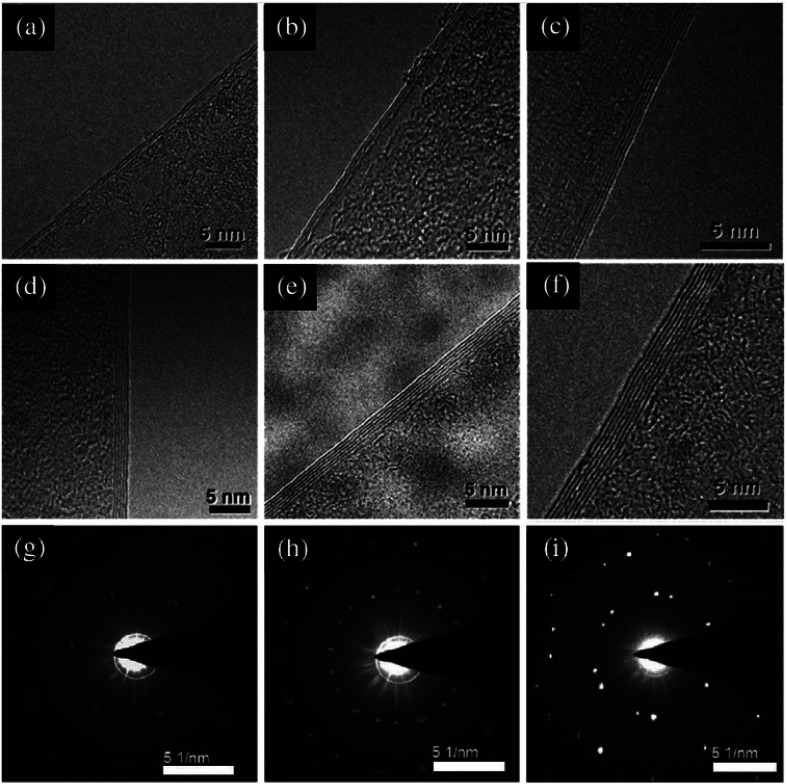
High-resolution TEM images of the edges of graphene with different numbers of layers: (a) bilayer, (b) trilayer, (c) four layers, (d) five layers, (e) six layers and (f) seven layers. The typical SAED images of bilayer, trilayer and five-layer graphene taken from the centre of the domains are shown in (g)–(i), respectively.

The structure of the synthesized GO was confirmed using the TEM image (Fig. 4), showing the GO layered structure.^94^ In addition, 2–7 graphene layers can be clearly seen in the TEM micrographs in Fig. 4(a)–(f). The SAED patterns shown in Fig. 4(g)–(i) are irregular, and the bilayer graphene, trilayer graphene and five-layer graphene films cannot be justified based on these patterns. Thus, other characterizations, such as Raman spectroscopy, are crucial to support the TEM results.

### 3.5. Selection of lung cancer biomarker

**Fig. 5:**
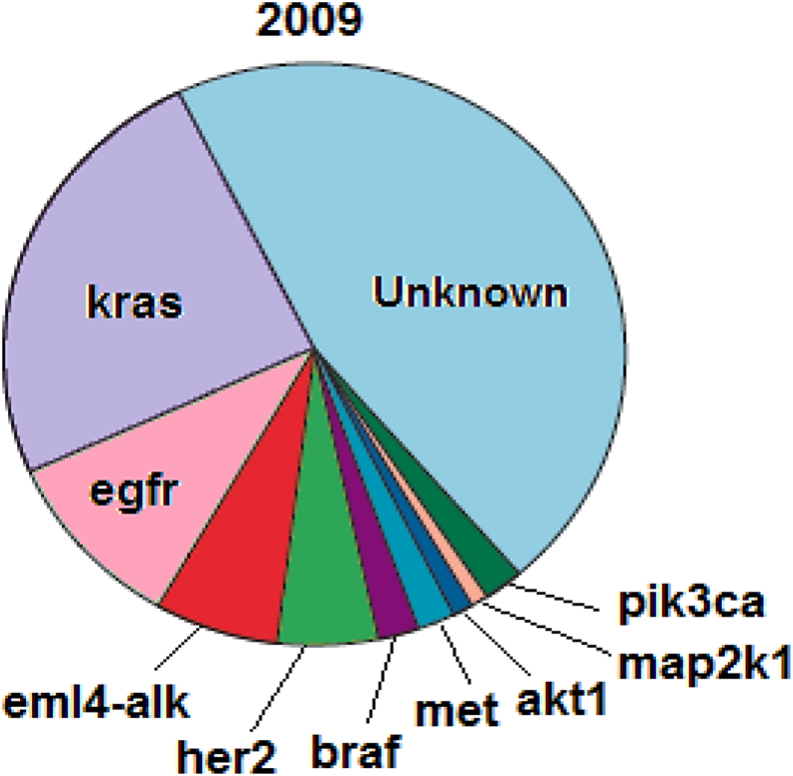
Biomarker genes typically present in NSCLC.

As one of the most prevalent cancer types worldwide, lung cancer is detected using various biomarkers, including exhaled air volatile organic compounds (VOCs), carcinogenic embryonic antigen (CEA), neuron-specific enolase (NSE), progastrin-releasing peptide (Pro GRP), cytokeratin-19 fragments (Cyfra21-1), squamous cell carcinoma antigen (SCCA), some miRNAs (*e.g.*, miR-155, miR-197, and miR-182), and some genes such as egfr, kras, *etc.* In addition to disease detection, some of these biomarkers are useful for examining disease progression, patients' response to treatment, and post-treatment disease recurrence.^51–53^ According to previous studies, lung cancer patients are prone to numerous gene mutations, a few of which are common among most patients. These common mutations include mutations in codons 12 and 13 of the kras gene, mutations in exons 21-18 of the egfr gene, mutations in exon 20 of the her2 gene, *etc.* William Pao and Nicolas Girard have recently investigated biomarkers genes typically present in NSCLC and introduced mutations in kras and egfr genes as respectively the most frequent mutations in patients with NSCLC, among other identified factors. The biomarker genes typically present in NSCLC (2009) are listed in Fig. 5.^54,95–97^

**Fig. 6:**
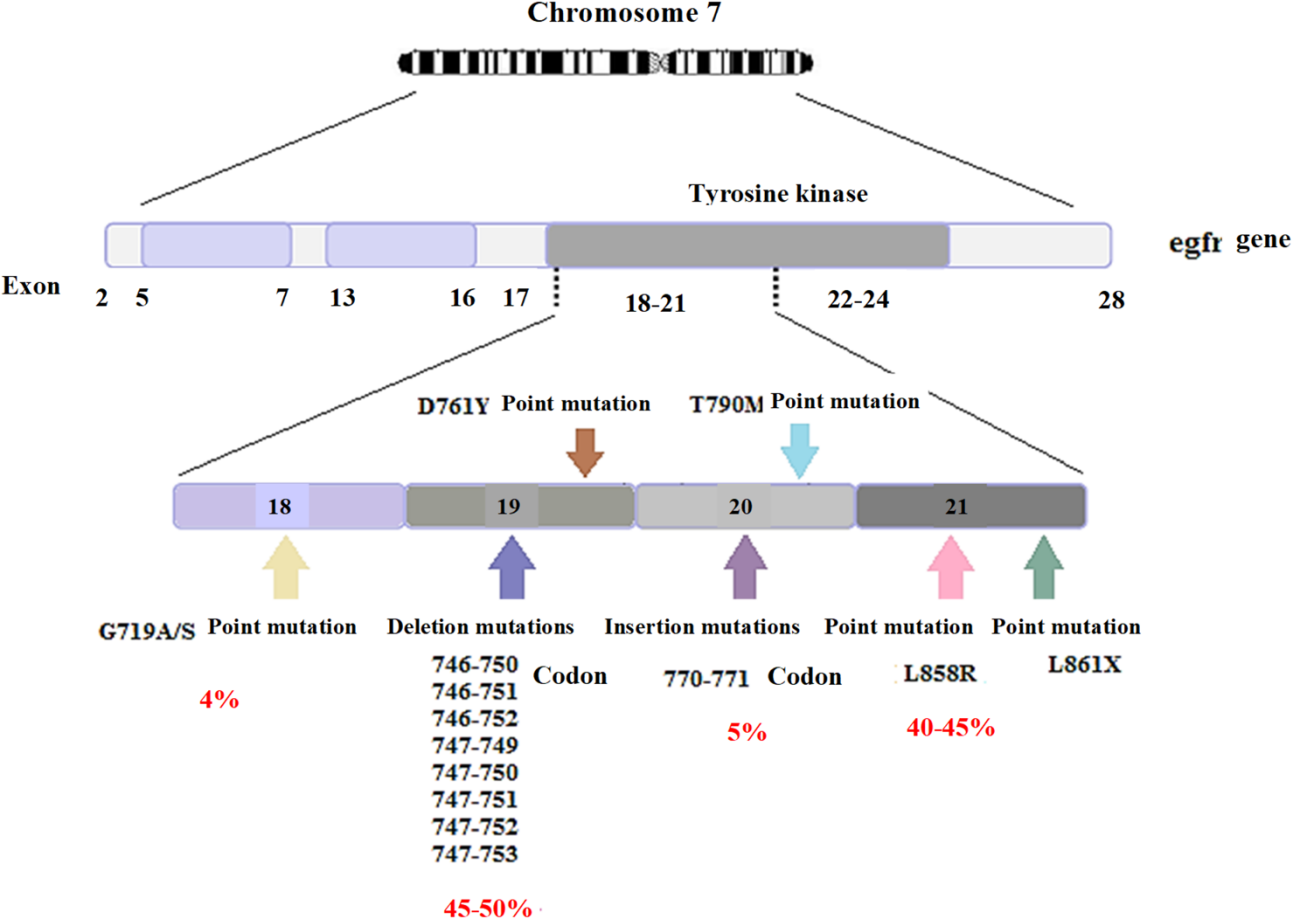
The frequencies of mutations in the egfr gene.

The egfr is an oncogene consisting of 28 exons with a length of 188 kbp. The frequent mutations in this gene include deletion, insertion, and point mutations, mainly located in the first four exons of the tyrosine kinase site. Some of these mutations include exon 19 deletion mutations including codons 746–753 (different codons are involved in deletion mutations in various patients; they include codons 746–750 in some patients and codons 747–752 in others). Besides, the L858R point mutation in exon 21, in which leucine is converted into arginine in codon 858, insertion mutations in exon 20, involving codons 770–771, and the G719 point mutation in exon 18, in which glycine is converted into serine, alanine, or cysteine in codon 719 are other examples of such mutations. Exon 19 deletion mutations and exon 21 L858R point mutation account for almost 90% of mutations in the egfr gene; in most cases, deletion mutations are more frequent than the L858R point mutation. Fig. 6 illustrates the frequencies of other mutations.^55–58^

NSCLC accounts for over 80% of lung cancer cases, and mutations in the egfr gene are highly frequent among gene mutations of the lung cancer cause. Among the mutations of this gene, exon 19 deletion mutations account for a high percentage. In this study, therefore, deletion mutations in exon 19 of the egfr gene (including codons 746–752) were selected as lung cancer biomarkers.

### 3.6. Interpretation of emission spectra

#### 3.6.1. Examination of the DNA probe fluorescence spectrum

**Fig. 7:**
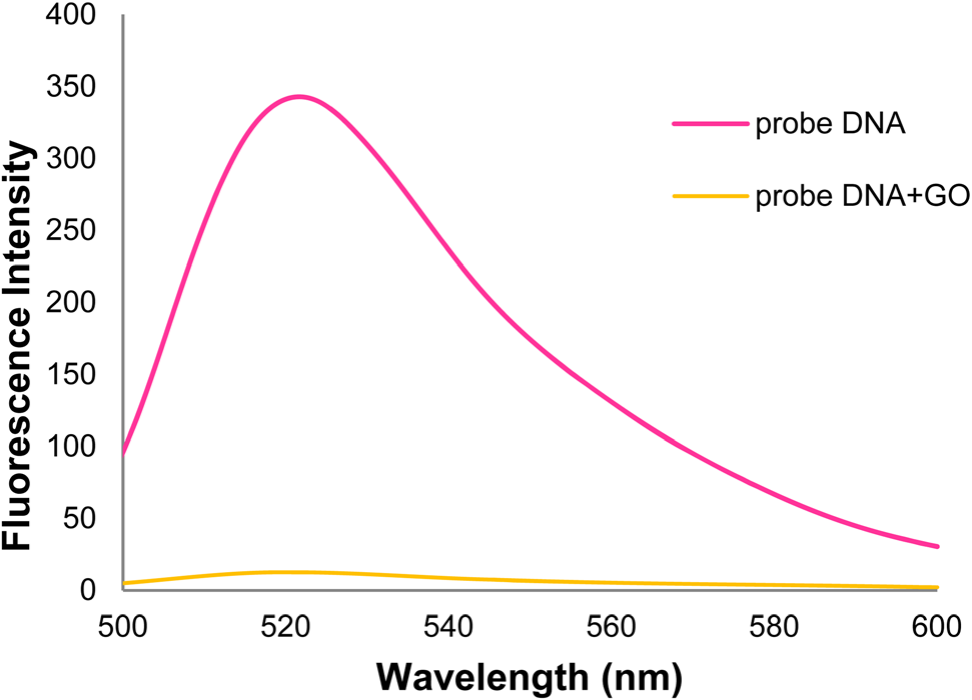
Fluorescence spectra of the DNA probe and GO + DNA probe.

The DNA probe oligonucleotide was labeled with the FAM fluorescent dye (carboxy fluorescein) and codons 746–752 in exon 19 of the egfr gene. As shown in the DNA probe fluorescence spectrum (Fig. 7), strong fluorescence emission is observed in the 520 nm wavelength in the absence of GO; however, the fluorescence intensity decreased with adding GO. The DNA probe fluorescence emission intensity decreased with >95% in the presence of GO after 32 min. This process is attributed to the DNA probe adsorption of the GO surface through non-covalent interactions (*e.g.*, π–π stacking) between the ring-type structures of nucleobases and hexagons of the GO aromatic lattice, hydrogen bonds between the –OH groups of GO, the –NH_2_ and –OH groups in the DNA probe, and van der Waals force.^59–61,98–100^

#### 3.6.2. Optimization of the DNA probe adsorption time on the GO surface

**Fig. 8:**
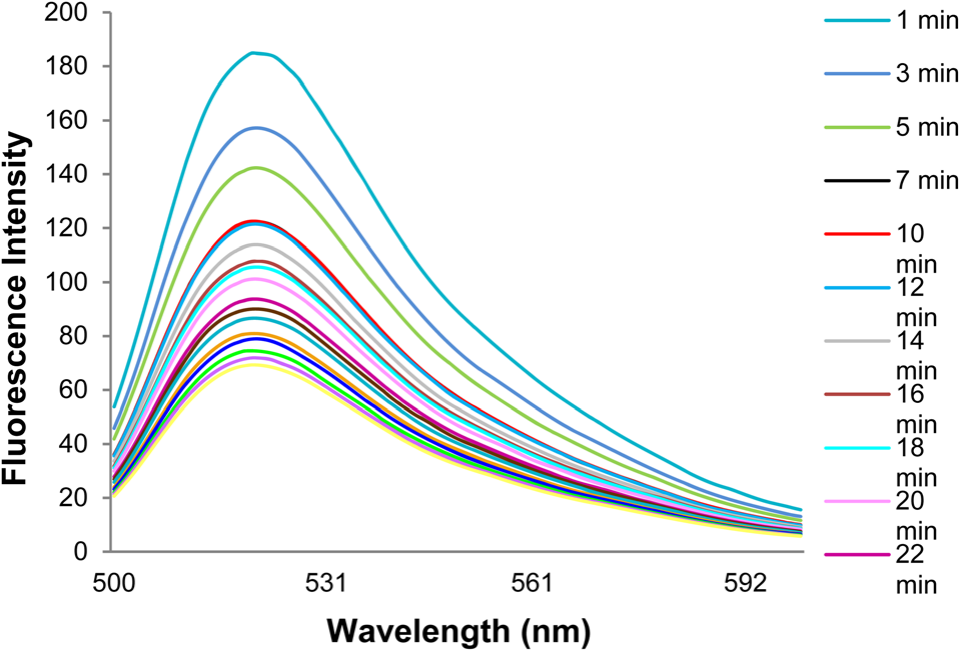
The DNA probe fluorescence spectra in the presence of GO at different times.

According to the DNA probe fluorescence spectra in the presence of GO at different times, the fluorescence emission intensity decreased gradually with the DNA probe adsorption on the GO surface over time. The DNA probe fluorescence intensity reached a constant value after 32 min, followed with obtaining the optimal time of the DNA probe adsorption on the GO surface. It should be mentioned that the excitation and emission wavelengths were 485 and 520 nm, respectively(Fig. 8).

**Fig. 9:**
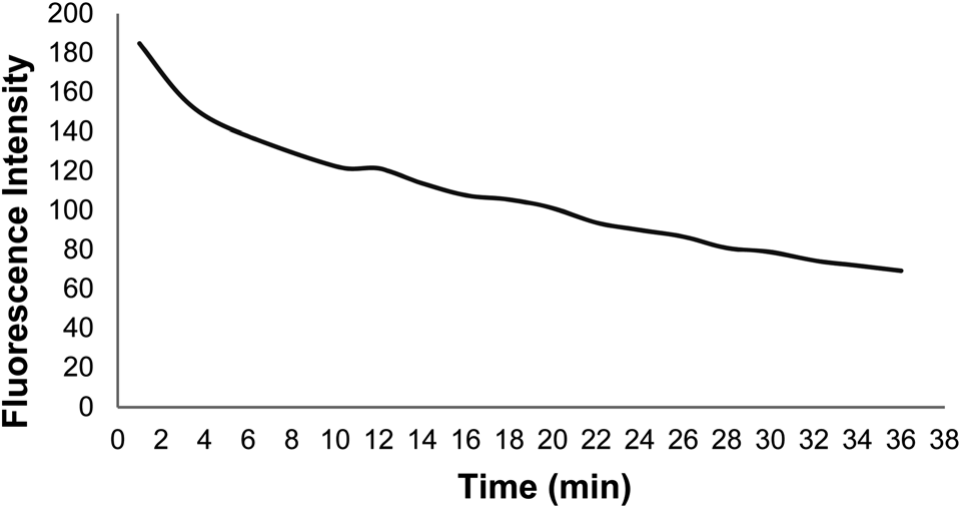
Changes in the GO + DNA probe fluorescence intensity at different times.

Fig. 9 shows changes in the DNA probe fluorescence intensity in the presence of GO at different times. An optimal time of 32 min was obtained for the DNA probe adsorption on the GO surface.

#### 3.6.3. Optimization of the GO dose in the presence of the DNA probe

**Fig. 10:**
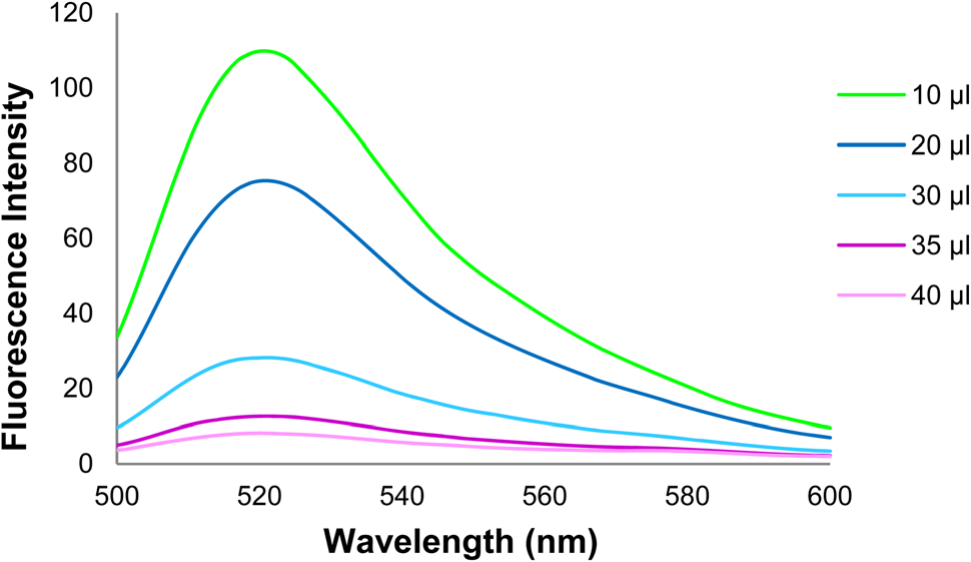
The DNA probe fluorescence intensity at different Go doses (1 mg ml^−1^).

**Fig. 11:**
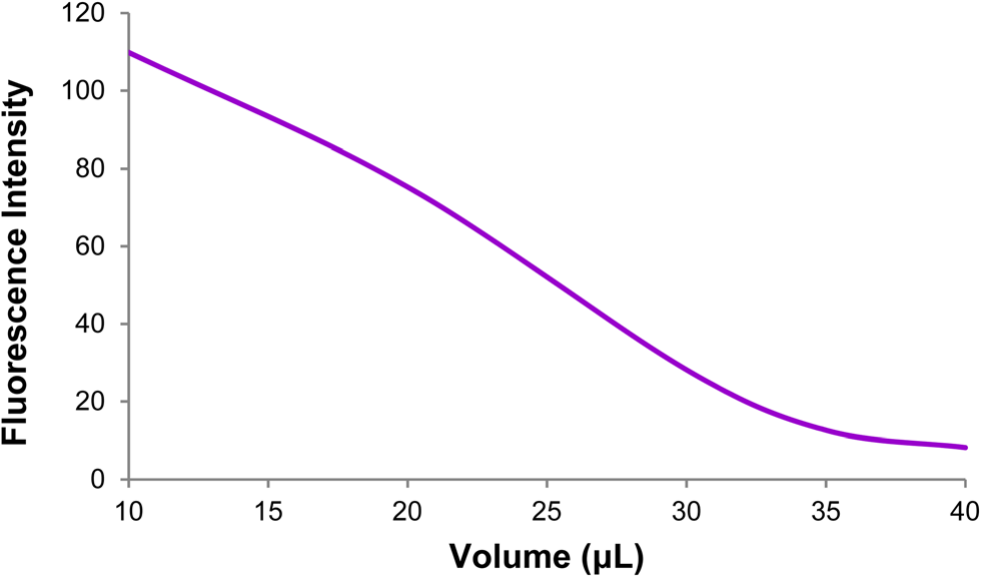
Changes in the GO + DNA probe fluorescence intensity at different GO doses (1 mg ml^−1^).

The effect of the GO dose on the DNA probe fluorescence emission intensity was examined in this step (Fig. 10 and 11). Based on the data, an increase in the GO dose increased the DNA probe adsorption on the GO surface, and the DNA probe fluorescence emission intensity decreased gradually in the 520 nm emission wavelength. Finally, an optimal GO dose of 35 μg was obtained per 100 pmol of the DNA probe.

#### 3.6.4. Examination of the DNA probe-GO + target (healthy) DNA fluorescence spectra

**Fig. 12:**
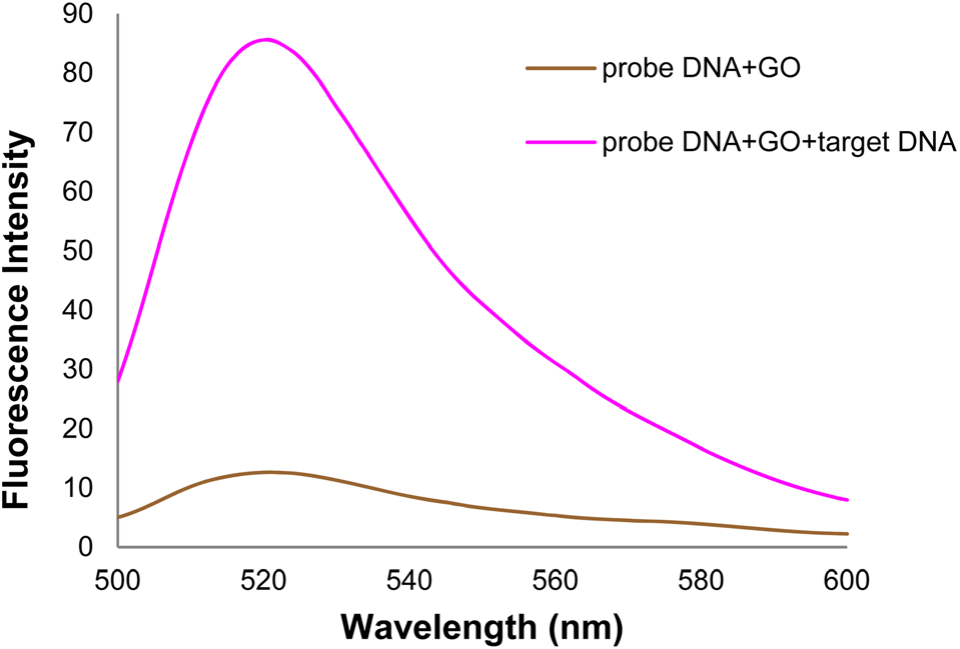
The fluorescence spectra of GO + DNA probe and target DNA + GO + DNA probe.

As shown in Fig. 12, the DNA probe-GO fluorescence emission intensity increased in the 520 nm emission wavelength with adding target (healthy) DNA. The DNA probe is hybridized with target DNA, and the resulting double-stranded DNA (dsDNA) is separated from the GO surface. When the DNA probe is hybridized with target DNA, nucleobases are protected in the phosphate backbone of dsDNA, which mostly negates the possibility of non-covalent interactions (π–π stacking) and the hydrogen bond. Unpaired nucleobases play an important role in DNA adsorption on the GO surface. Thus, dsDNA is adsorbed on the GO surface at a very lower level than single-stranded DNA.^62,90^

#### 3.6.5. Optimization of the target DNA and DNA probe hybridization time in the presence of GO

**Fig. 13:**
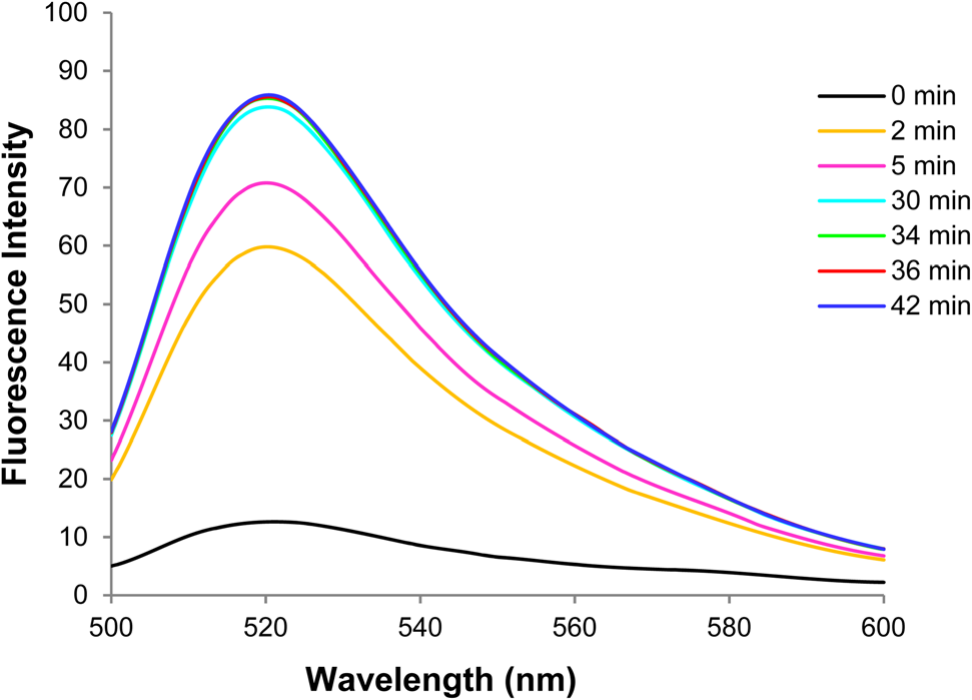
The fluorescence spectra of GO-DNA probe + target DNA at different times.

According to the fluorescence spectra of the GO-DNA probe complex in the presence of target (healthy) DNA at different times, the fluorescence emission intensity increased gradually with the target DNA and DNA probe hybridization over time and then reached a constant value after 34 min. Thus, the optimal time of target DNA and DNA probe hybridization was obtained in the presence of GO(Fig. 13).

**Fig. 14:**
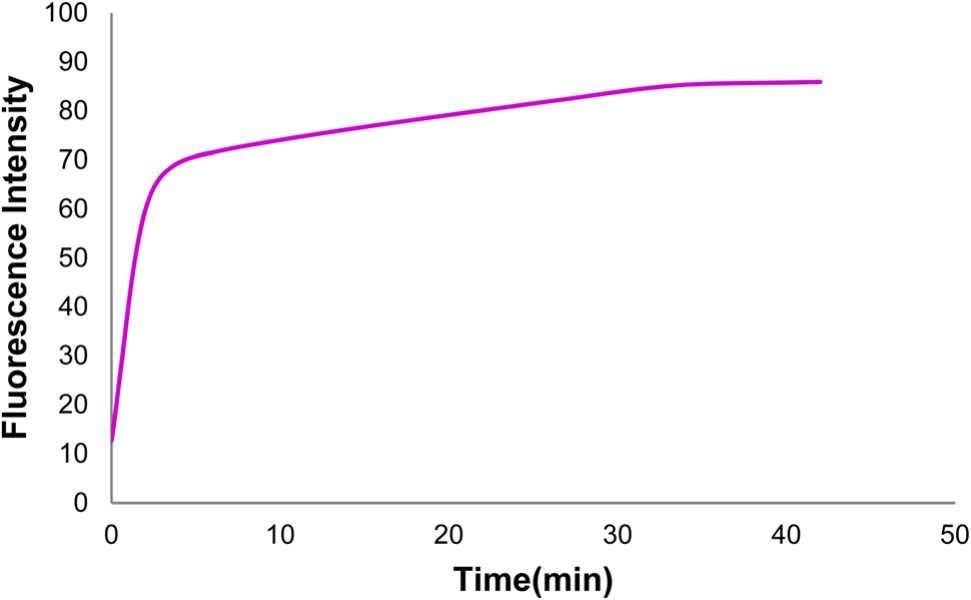
Changes in the GO-DNA probe fluorescence intensity in the presence of target DNA at different times.

The optimal hybridization time of target (healthy) DNA and the DNA probe was determined at 34 min with drawing the curve of changes in the GO-DNA probe fluorescence intensity in the presence of target DNA at different times (Fig. 14).

#### 3.6.6. Examination of changes in the GO-DNA probe fluorescence intensity at different concentrations of target DNA

**Fig. 15:**
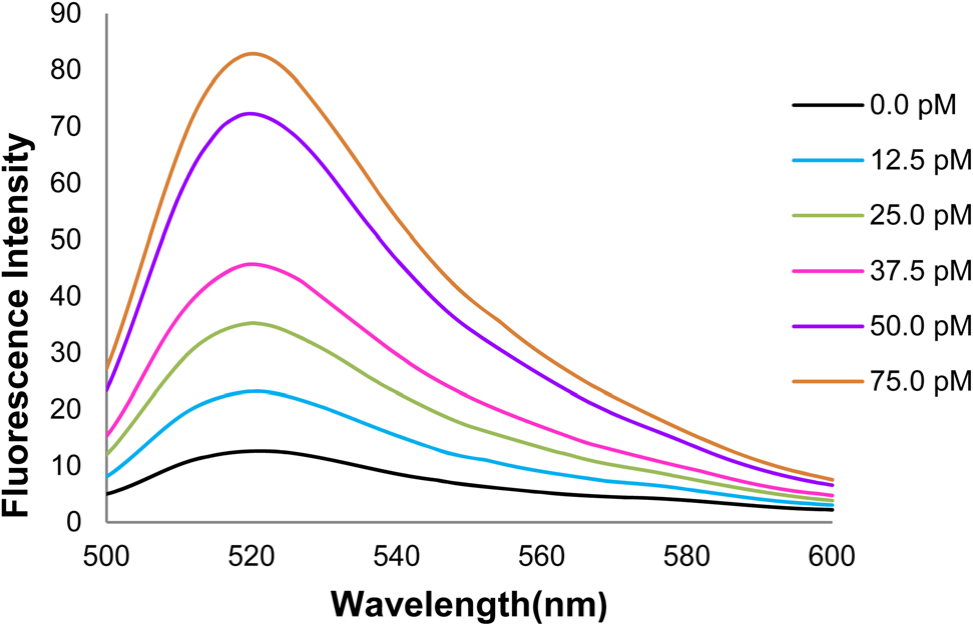
The GO-DNA probe fluorescence spectra at different concentrations of target DNA.

According to the effect of target (healthy) DNA concentrations on the GO-DNA probe fluorescence intensity, an increase in the target DNA concentration in the 520 nm emission wavelength led to a gradual increase in fluorescence intensity(Fig. 15).

**Fig. 16:**
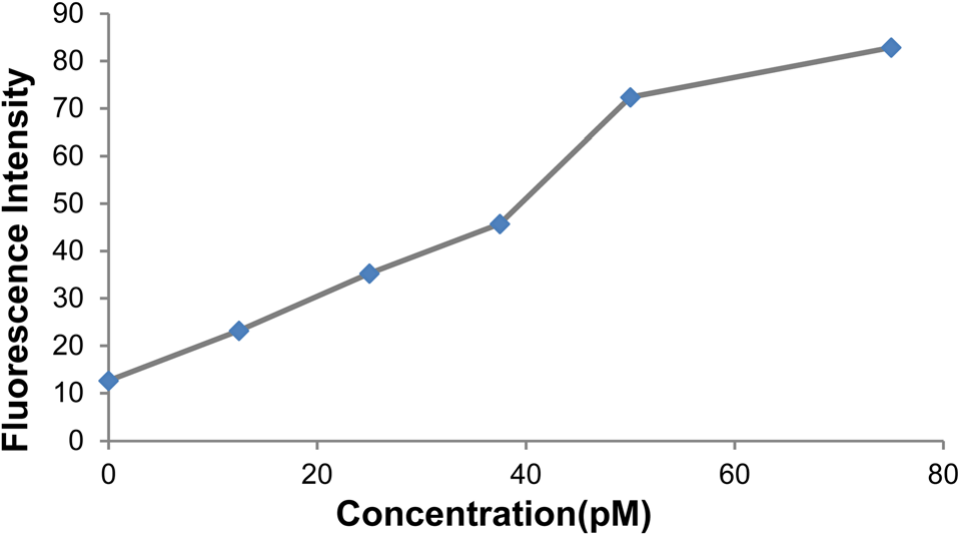
Changes in the GO-DNA probe fluorescence intensity at different concentrations of target DNA.

**Fig. 17:**
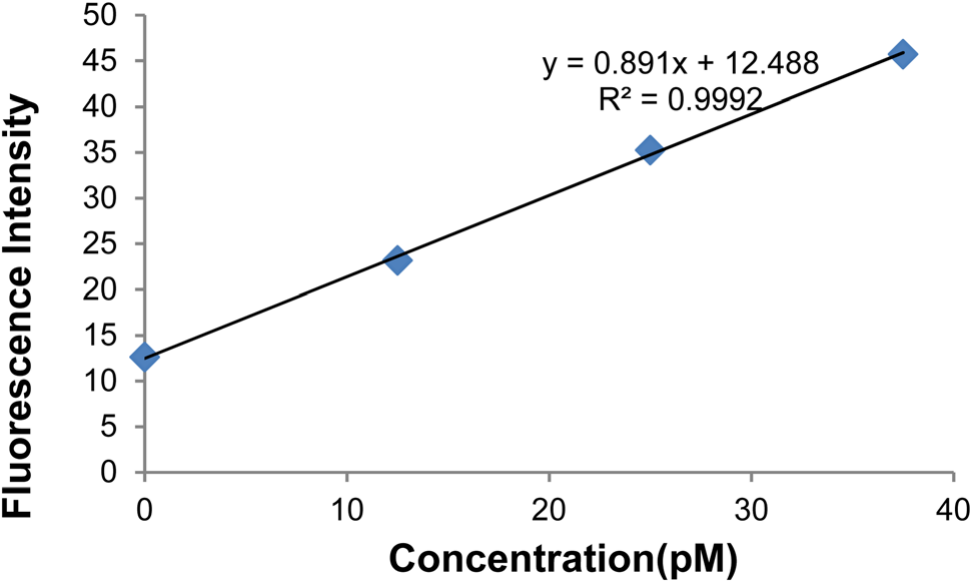
Changes in the GO-DNA probe fluorescence intensity at target DNA concentrations < 40 pmol.

Fig. 17 depicts the curve of changes in the GO-DNA probe fluorescence emission at different concentrations of target DNA (0–40 pmol). According to Fig. 16 and 17, this method can be used to determine the concentrations of target DNA (from 0 to 40 pmol) in unknown samples.

#### 3.6.7. Examination of the GO-DNA probe fluorescence spectra in the presence of (mutated) mDNA

**Fig. 18:**
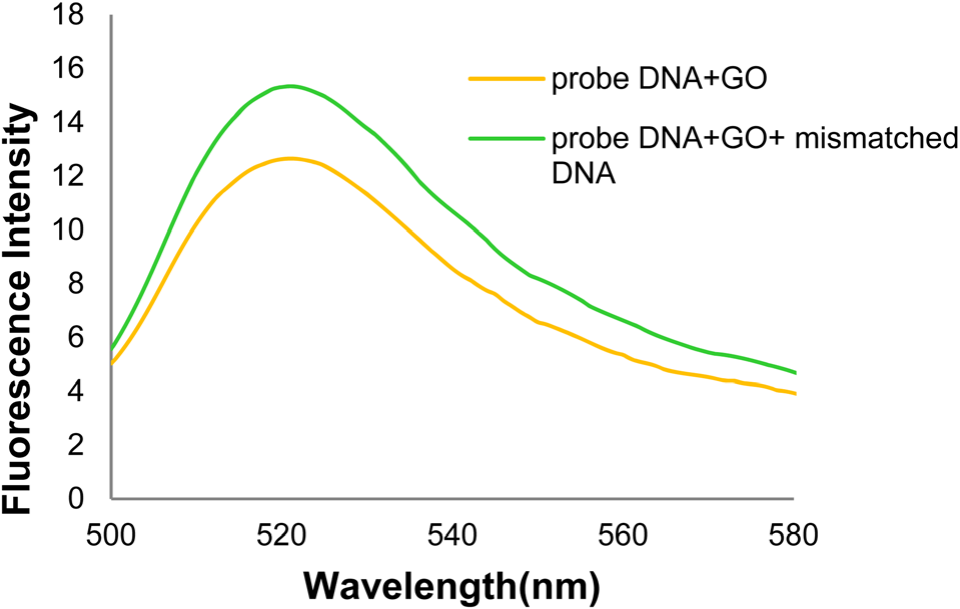
The fluorescence spectra of the GO-DNA probe and GO + DNA + mDNA probe.

Fig. 18 displays the GO-DNA probe fluorescence spectra in the presence of (mutated) mDNA. Since a complementary sequence of the DNA probe is absent in mDNA due to a deletion mutation, it was not hybridized with the DNA probe, leading to no formation of a dsDNA. Thus, the fluorescence intensity showed no effective changes in the 520 nm emission wavelength.

### 3.7. Lung cancer detection

**Fig. 19:**
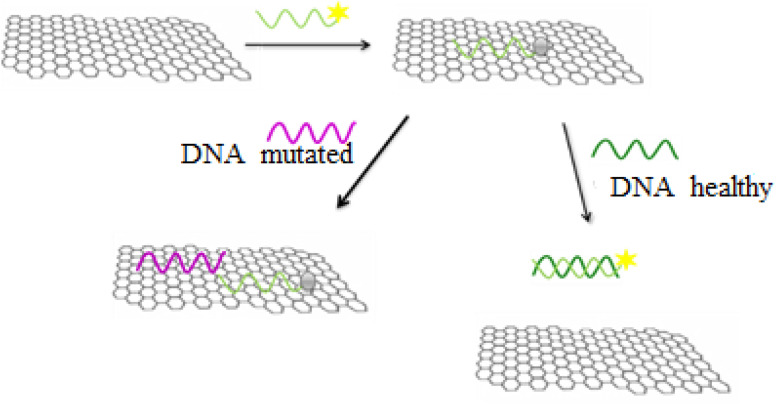
The Nano biosensor responses to target (healthy) DNA and (mutated) mDNA.

In the general procedure of lung cancer detection using the nanobiosensor (Fig. 19), the DNA probe is adsorbed on the GO surface, and the fluorescence intensity increases with adding target (healthy) DNA. However, the fluorescence intensity shows no changes with adding (mutated) mDNA. In other words, the designed nanobiosensor responds differently to the two (healthy and mutated) DNAs, making it possible to detect mDNA (in cancer patients).

**Fig. 20:**
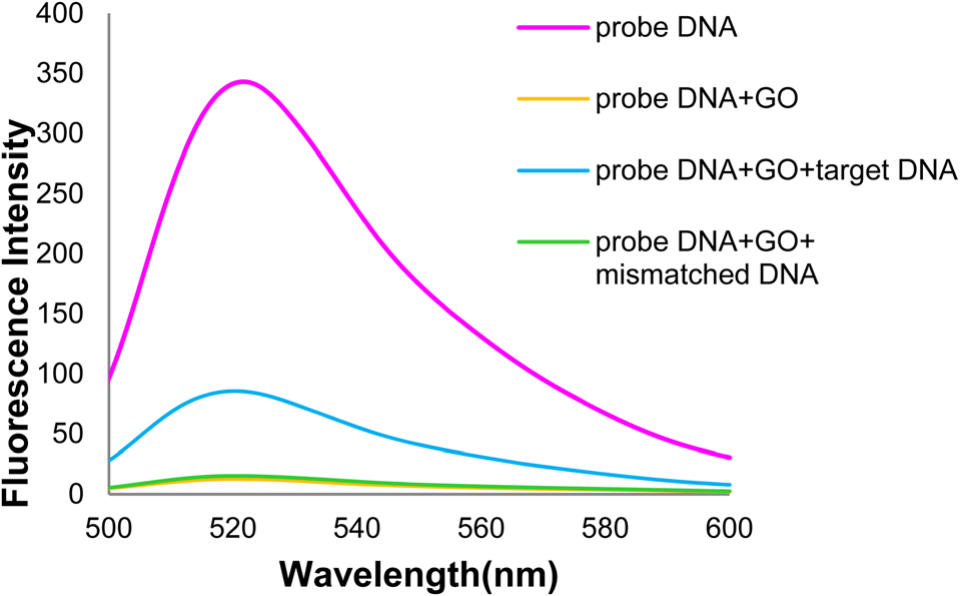
Comparison of GO-DNA probe fluorescence intensity in the presence of two (healthy and mutated) DNAs.

Fig. 20 shows the GO-DNA probe fluorescence spectra, indicating the different DNA probe fluorescence intensity in the presence of two (healthy and mutated) DNAs. Accordingly, the incidence of deletion mutations in codons 746–752 of the egfr gene as the lung cancer biomarker is examined and detected in the DNA of interest.

## 4. Conclusion and outlook

1. Since graphene oxide (GO) is readily available and exhibits exceptional optical, electrical, mechanical and chemical properties, it has attracted increasing interests for use in GO-DNA based sensors. In solution, graphene oxide is as an excellent acceptor of fluorescence resonance energy transfer (FRET) to quench the fluorescence in dye labeled DNA sequences. The application of the electrochemical GO-DNA based sensors is also summarized because graphene oxide possesses exceptional electrochemical properties. GO-DNA based sensors perform well at low cost, and high sensitivity, and provide low detection limits. Additionally, GO-DNA based sensors should appear in the near future as scientists explore their usefulness and properties. Finally, future perspectives and possible challenges in this area are outlined. The results of these recent research studies exhibit the outstanding performance of graphene oxide compared with current techniques. However, some challenges related to DNA sensors-based graphene oxide remain and need to be resolved. Because ssDNA is adsorbed on the surface of graphene oxide, not all dsDNA can detach from the surface of graphene oxide after the complementary ssDNA, protein or other molecules combine to ssDNA. This hinders the further improvement of the sensitivity of reported DNA sensors based on most of the recent publications reviewed, although a few authors have reported some methods for solving this problem. Currently, most published literature reports that only one target can be detected for one DNA-based sensor using graphene oxide in the liquid phase. Graphene oxide in the solid phase was scarcely explored. If more targets can be detected with one sensor, the throughput of detection will be improved. Graphene oxide bears oxygen functional groups on its basal planes and edges. Therefore, graphene oxide in the solid phase can also be used to make devices for sensing without chemical modification on the surface of graphene oxide. The devices are made using lithography, thermal evaporation and other micro–nano related scientific technology. If one GO–DNA based sensor is like an array with different DNA elements, many targets will be detected.

2. In the present research, a GO-DNA-based Nano biosensor was proposed for lung cancer detection. Graphene oxide was synthesized using the Hummers' method, and its structure was examined and confirmed using FT-IR, UV-vis, and TEM images. The adsorption of a FAM-labeled DNA probe on graphene oxide in the presence of target (healthy) DNA and (mutated) mDNA was then investigated using fluorescence spectroscopy. The different responses of the nano biosensor to healthy and mutant DNAs allowed for lung cancer detection. Relying on nanotechnology, therefore, lung cancer can be detected through fast, easy, and cost-effective methods.

## Conflicts of interest

The author declare that they have no competing interests.

## Supplementary Material
